# Thyroid Function, Urinary Iodine, and Thyroid Antibody Status Among the Tribal Population of Kashmir Valley: Data From Endemic Zone of a Sub-Himalayan Region

**DOI:** 10.3389/fpubh.2020.555840

**Published:** 2020-10-28

**Authors:** Mohd Ashraf Ganie, Bashir A. Charoo, Tajali Sahar, Moomin Hussain Bhat, Sheikh Abid Ali, Madiha Niyaz, Shivani Sidana, Arajmand Yaseen

**Affiliations:** ^1^Department of Endocrinology, Sheri Kashmir Institute of Medical Sciences, Srinagar, India; ^2^Department of Pediatrics, Sheri Kashmir Institute of Medical Sciences, Srinagar, India; ^3^Department of Clinical Biochemistry, Sheri Kashmir Institute of Medical Sciences, Srinagar, India

**Keywords:** thyroid dysfunction, urinary iodine, hypothyroidism, Kashmir, tribal population

## Abstract

**Background:** There are scarce data on the prevalence of thyroid disorders and urinary iodine status among tribal populations of India, with no reported data from Kashmir valley.

**Objectives:** To estimate the prevalence of thyroid disorders and evaluate urinary iodine concentration (UIC) and thyroid autoantibody status among Gujjar and Bakerwal tribes of Kashmir valley.

**Methods:** This cross-sectional study recruited tribal subjects using multistage cluster sampling from 5 out of 22 districts of Jammu and Kashmir (J&K). Using a predesigned questionnaire, the details of past or current medical history and drug intake, including thyroid hormone medication etc. were recorded after obtaining an informed consent. Examination included anthropometry (height, weight, waist circumference), blood pressure measurement, and relevant general physical examination focusing on goiter palpation, while as laboratory assessment included estimation of serum thyroid hormone levels, antithyroid peroxidase antibody (anti-TPO Ab), and urinary iodine concentration.

**Results:** A total of 763 subjects (56.4% women and 43.6% men) with a mean(±SD) age of 39.46 (±17.51) ranging from 10 to 85 years and mean(±SD) body mass index (BMI) of 21.28 (±4.16) kg/m^2^ were studied. Goiter was detected in 6.8%, while 33.2% subjects had some form of thyroid dysfunction (including 24.1% subclinical and 6.8% overt hypothyroidism). Subclinical and overt hyperthyroidism were observed in 1.3 and 0.9% of cases, respectively. Anti-TPO Ab was elevated in 13.6%, while the median [interquartile range (IQR)] for UIC was 154.50 (135) μg/L [156.13 (134) μg/L in men and 147.26 (136) μg/L in women]. A negative correlation was observed between UIC and anti-TPO Ab (*r* = −0.087, *P* = <0.05).

**Conclusion:** These novel data on iodine and thyroid status among a tribal population of India generally inhabiting in remote sub-Himalayan belts demonstrate high prevalence of subclinical hypothyroidism (SCH) with persistent iodine deficiency. These preliminary data may warrant large well-designed studies to carry out comprehensive assessment of the problem in this high-risk and marginalized population.

## Introduction

Thyroid disorders are highly prevalent endocrine diseases, with iodine deficiency remaining a common cause of thyroid dysfunction worldwide. Of the cumulative 108 million people with endocrine and metabolic disorders in India, 42 million suffer from various thyroid disorders (1). The prevalence of hypothyroidism in India is more than that reported in developed countries (2). Data from eight different cities in India reveal a prevalence of hypothyroidism as 10.9% with higher prevalence of hypothyroidism among cities located inland than the coastal ones (3), which is attributable to consumption of sea food rich in iodine (11.7 vs. 9.5%) in the latter ones. However, despite the effective implementation of universal salt iodization since 1983, the prevalence of hypothyroidism is not declining. Jammu and Kashmir (J&K), the northern most region of India located in the Himalayan belt, has been a known iodine-deficient endemic zone among other hilly regions of India (4). Preliminary epidemiological data generated in 1995 demonstrated a high prevalence of goiter with very low urinary concentration (UIC) among school children from the general population (5). A follow-up data in the same region 15 years later demonstrated a significant drop in the goiter prevalence, indicating effective implementation of the National Iodine Deficiency Disorders Control (NIDDC) program (6). There are indicators that the transition from iodine deficient to replete state is followed by a spurt in autoimmunity and autoimmune thyroid disorders (7). Although an easy-to-detect and inexpensive-to-treat disease, patients with hypothyroidism in India often remain undetected and untreated, leading to impairment in work performance and economic productivity of people (8). The tribal population of the country, in general, and from J&K, in particular, resides on the inaccessible, mountainous regions, which are traditionally endemic iodine-deficient areas. There are scarce data on the incidence and prevalence of thyroid disorders in the tribal population of India except small studies on incidence of iodine deficiency from Orissa and Kerala (9, 10). No such studies have been conducted in the tribal population of J&K, which comprises around 10.9% of the country's total tribal population. Hence, the present study, part of a larger survey on tribal health, aimed to assess the prevalence of thyroid dysfunction, and antibody and urinary iodine status of this subpopulation.

## Materials and Methods

The present health survey enrolled indigenous tribal population (Gujjar and Bakerwal) across different districts of the Kashmir valley. The study was carried out jointly by All India Institute of Medical Sciences (AIIMS), New Delhi and Sheri-Kashmir Institute of Medical Sciences (SKIMS), Kashmir. The study protocol was approved by the SKIMS Institutional Ethics Committee. The substudy involved evaluation of thyroid function, antithyroid peroxidase (anti-TPO) levels, and UIC of the subjects, while the rest of the data are presented elsewhere. Since there was paucity of any available reports regarding prevalence of hypothyroidism among tribal population, the sample size was calculated based on the prevalence of hypothyroidism among tribal populations elsewhere in India.

### Subject Selection

Out of the 22 districts of J&K housing ~1.49 million tribals, we selected five districts (Anantnag, Pulwama, Ganderbal, Kupwara, and Srinagar) based on the multistage cluster sampling with probability proportional to size (PPS) technique. From each district, a tehsil (administrative subdivisions of district), from each tehsil, a block (smaller administrative subdivision) was selected, and from each block, two villages were randomly selected. To facilitate greater public participation and acceptance, liaising with village heads, religious leaders, local health workers of government health agencies, non-government organizations etc. was initiated at the outset. Information brochures and pamphlets detailing the study objectives and procedures were circulated for the purpose of general awareness among the study subjects. Eligible subjects of all age groups were asked to sign an informed consent. In case of subjects <18 years, written consent was obtained from their parents after they furnished an assent. The subjects who were physically or mentally challenged or were taking thyroid medications or iodine-containing supplements or who refused consent were excluded from the study. Women who were currently pregnant were also not included in the study.

### Data Collection

On the day of the study, the research team along with other supportive staff visited the households as per prior mapping of the households of the village. All the family members of the households were invited, except those fulfilling exclusion criteria. A modified WHO-STEPS questionnaire was administered for capturing data regarding the sociodemographic indices, medical facts, dietary intake, etc. via a face-to-face interview with the subjects, preferably in the local language. All medical facts including history of any current or past sickness, relevant family history, and drug intake with emphasis on thyroid-related diseases or medications were recorded. The questionnaire was duly validated by pretesting in 100 subjects to ensure suitability and comprehension in this subset of population.

Anthropometric measurements of the study participants including weight, height, and waist circumference were carried out by using standardized methodology. A digital body weight scale with a range of 0.1–200 kg and a stadiometer (Seca-213, Hamburg Germany) were used for weight and height measurements, respectively. Blood pressure (BP) was taken as a mean of three readings as measured by a physician on the left arm of each study subject in sitting position, using a cuff of appropriate size at the level of the heart (Omron 714, Kyoto Japan). Goiter assessment was done by a trained and experienced endocrinologist (MAG) using palpation method as per latest WHO grading (grade 0, no goiter; grade 1, thyroid palpable but not visible; and grade 2, thyroid visible with the neck in normal position).

### Laboratory Evaluation

After capturing clinical details, all the subjects who furnished informed consent were subjected to random blood sampling by qualified phlebotomists. A 10-ml venous blood sample was drawn for measurement of biochemical parameters and thyroid function tests. The blood samples were allowed to settle for 15 min at room temperature before they were centrifuged at 1,100 *g* (Eppendorf 5804R, Hamburg, Germany) for 10 min at the study site, and the serum samples were transported in cold boxes (~4°) to the storage site (SKIMS) where samples were stored at −20°C until the assay. A sterile plastic urine container was used to collect the morning first-catch urine sample of all the subjects for estimation of urinary iodine and creatinine. Biochemical estimations were performed using an automatic analyzer (Response 920, DIASYS Germany), and thyroid-stimulating hormone (TSH), thyroxine (T4), and tri-iodothyronine (T3) were estimated by immunoassay analyzer (Beckman Coulter Inc. USA). Anti-TPO Ab assay was performed using an electrochemiluminescence immunoassay (ECLIA) analyzer (Cobas e411, Roche Diagnostics, CA, USA). The reference ranges of TSH (0.45–6.5 μIU/ml), T4 (0.5–12 μg/dl), T3 (0.7–2.5 ng/ml), and anti-TPO Ab (0–34 IU/L) were taken as per kit normal. Accordingly, subclinical hypothyroidism (SCH) was diagnosed if serum T4 levels were normal and serum TSH elevated (>6.5 μIU/ml), while subclinical hyperthyroidism was diagnosed if TSH was low (<0.45 μIU/ml) with presence of normal serum T4 concentrations. Overt hypothyroidism was diagnosed if T4 is low (<4.5 μg/dl) with presence of high TSH and overt hyperthyroidism by high T4 level (>12 μg/dl) and low TSH level (<0 1 μIU/ml). UIC was estimated using Sandell–Kolthoff reaction based on the principle that iodide catalyzes the reduction in ceric ammonium sulfate (yellow) to cerous form (colorless), detected by rate of color disappearance. Subjects were categorized on the basis of UIC levels as mild (50–99 μg/dl), moderate (30–49 μg/dl), and severely deficient (<30 μg/dl). Participants with UIC between 100 and 299 μg/dl were labeled as iodine-sufficient group, and those with ≥300 μg/dl were labeled as iodine excess group.

### Statistical Analysis

The data were entered in Microsoft excel and analyzed using IBM-SPSS version 21.0 (SPSS Inc. Chicago, IL, USA). Categorical variables were expressed as counts and percentages and compared using the chi-square test. An independent Student's *t-*test and one-way ANOVA with least significant difference (LSD) *post hoc* test were used for the comparison of two and more than two groups, respectively. The normality of data was assessed using Kolmogrov–Smirnoff test. Data were log transformed wherever necessary. Correlation was calculated using the Pearson's correlation method. A multivariate logistic regression analysis was applied to predict the risk factors for thyroid dysfunction. A two-tailed *P* < 0.05 was considered statistically significant.

## Results

A total of 763 healthy subjects (430 women and 333 men) were evaluated, had a mean(±SD) age of 39.46 ± 17.51 years (range, 10–85 years) with mean(±SD) body mass index (BMI) of 21.28 ± 4.16 kg/m^2^ (range, 22–39). Mean (±SD) systolic and diastolic blood pressures were 121.73 ± 17.88 and 79.82 ± 12.07 mmHg, respectively. Demographic characteristics are mentioned in Table 1.

**Table 1:** Description of clinical and biochemical parameters and urinary iodine estimation.

**Variables**	**Overall** **Mean ± SD** **(Min, Max)**	**<18 years** **Mean ± SD** **(*n =* 95)**	**18–60 years** **Mean ± SD** **(*n =* 580)**	**>60 years** **Mean ± SD** **(*n =* 88)**
Age (years)	39.46 ± 17.51 (10, 85)	15.50 ± 1.29	38.16 ± 12.17	71.57 ± 6.18
Weight (kg)	55.12 ± 11.73 (25, 95)	48.25 ± 10.27	56.65 ± 11.09	55.83 ± 11.60
Height (cm)	160.93 ± 11.09 (110, 189)	160.00 ± 11.77	161.64 ± 10.32	161.28 ± 10.94
BMI (kg/m^2^)	21.28 ± 4.16 (22, 39)	18.66 ± 1.94	21.72 ± 4.07	21.57 ± 4.16
Waist (cm)	80.06 ± 12.95 (55, 119)	68.25 ± 8.88	81.28 ± 13.02	80.02 ± 12.64
Hip (cm)	88.80 ± 11.30 (60, 119)	77.50 ± 11.82	89.97 ± 11.35	88.72 ± 10.04
Systolic blood pressure (mm Hg)	121.73 ± 17.88 (87, 180)	111.25 ± 2.50	120.54 ± 16.23	134.93 ± 20.79
Diastolic blood pressure (mm Hg)	79.82 ± 12.07 (51, 120)	72.50 ± 5.0	79.35 ± 11.53	87.91 ± 12.06
Blood glucose random (mg/dl)	117.49 ± 22.15 (71, 273)	93.25 ± 12.97	119.06 ± 23.64	120 ± 23.56
Blood glucose fasting (mg/dl)	104.55 ± 12.39 (73, 126)	88.00 ± 8.91	100.00 ± 9.87	106.40 ± 8.76
Serum T3 (ng/ml)	1.29 ± 1.10 (0.46, 8)	3.34 ± 3.04	1.18 ± 0.52	1.11 ± 0.28
Serum T4 (μg/dl)	7.25 ± 1.91 (2, 13)	7.92 ± 1.91	7.46 ± 1.43	8.42 ± 1.62
Serum TSH (μIU/ml)	4.62 ± 5.02 (0.03, 40.33)	4.06 ± 0.96	7.17 ± 6.18	6.74 ± 10.29
Anti-TPO antibody (IU/ml)	27.15 ± 44.74 (1, 277)	14.25 ± 17.19	32.77 ± 71.89	15.45 ± 18.94
Urine iodine (μg/L) [median(IQR)]	154.50 (135)	155.60 (110)	154.54 (135)	154.50 (153)

### Thyroid Dysfunction

**Figure 1:**
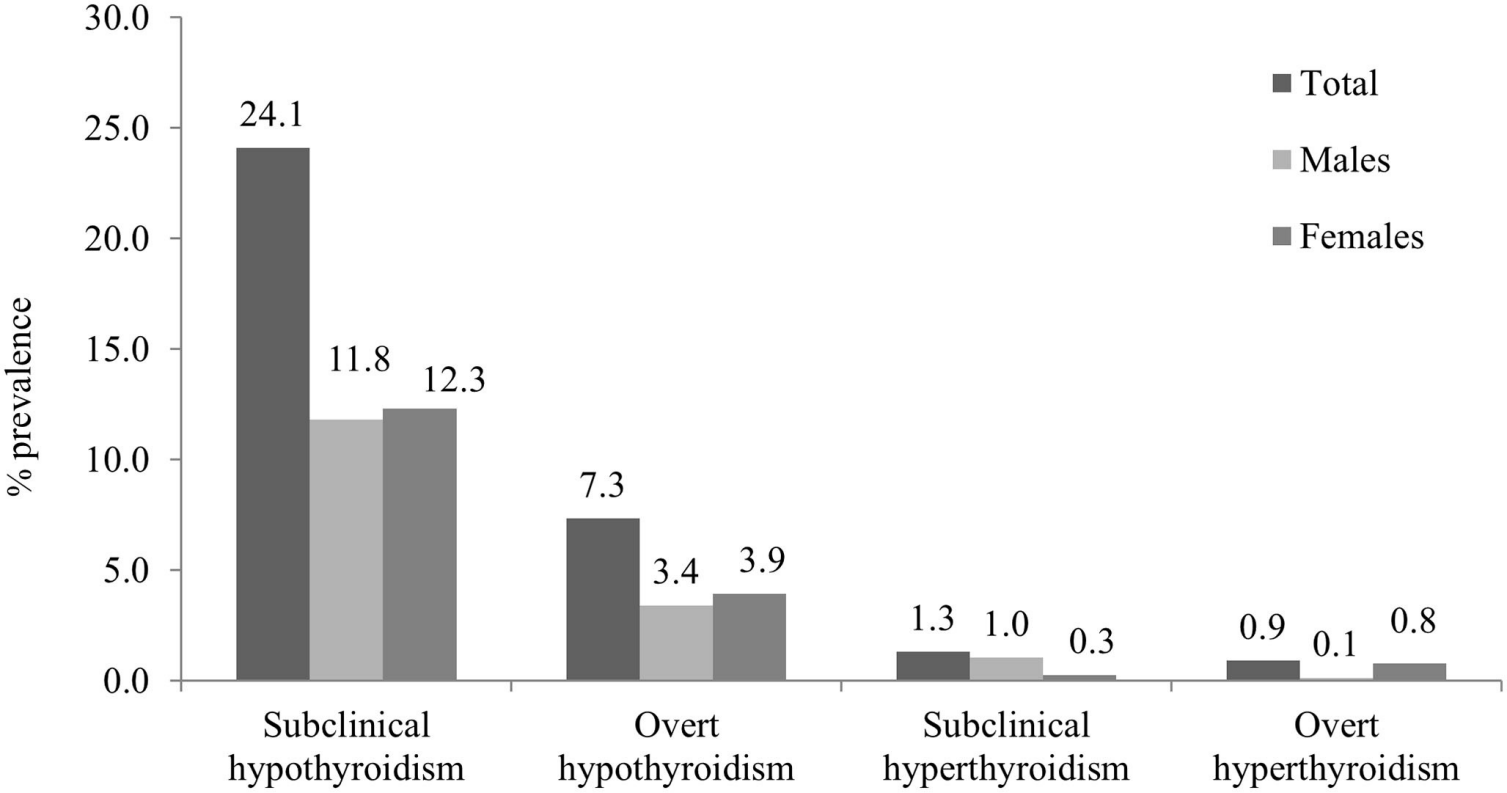
Overall and gender-wise prevalence of thyroid dysfunction.

Overall thyroid dysfunction was seen in 33.2% of the subjects (36.4% male and 30.5% female), with SCH being the most common form of thyroid dysfunction affecting 24.1% subjects (26.2% in men and 22.4% in women) (Table 2). Overt hypothyroidism was present among 6.8% of subjects (7.6% in men and 6.2% women). Subclinical hyperthyroidism was present in 1.3% and overt hyperthyroidism in 0.9% of studied subjects (Figure 1). The prevalence of hypothyroidism and hyperthyroidism was not significantly different between male and female subjects (*P* = 0.25). Overt hypothyroidism was significantly higher in the age group of 18–60 years (*P* = 0.03), whereas SCH was equally prevalent among subjects >60 years of age. Subclinical hyperthyroidism was also more common in the elderly (>60 years) (Table 2).

**Table 2:** Prevalence of various thyroid disorders among age categories.

	**<18 years**	**18–60 years**	**>60 years**	**Overall**
	**n (%)**	**n (%)**	**n (%)**	**n (%)**
n (%)	88 (11.5)	586 (76.8)	89 (11.7)	763 (100)
Overt hypothyroidism	4 (4.6)	44 (7.6)	4 (4.5)	52 (6.9)
Subclinical hypothyroidism	16 (18.4)	144 (24.5)	22 (25)	184 (24.1)
Euthyroid	68 (77)	385 (65.7)	59 (65.9)	510 (66.8)
Subclinical hyperthyroidism	0 (0.0)	7 (1.2)	3 (3.4)	10 (1.3)
Overt hyperthyroidism	0 (0.0)	6 (1.0)	1 (1.1)	7 (0.9)

Goiter prevalence was detected in 6.9% of the subjects, and according to WHO classification, 3.4% had grade 1, whereas 2.6% had grade 2, and 0.8% had grade 3 goiter.

### Thyroid Autoimmunity

Anti-TPO Ab was positive among 13.6% with antibody positivity equally distributed among men and women, but severity was noted more among women. As the age increased, the percentage of antibody positivity increased. Mild elevation (defined as 34–75 IU) was noted in 4.5% of male and 2.9% in female subjects, while 3.9% of female and 2.3% of male subjects were strongly anti-TPO Ab positive (defined as >75 IU).

### Urinary Iodine Excretion

**Figure 2:**
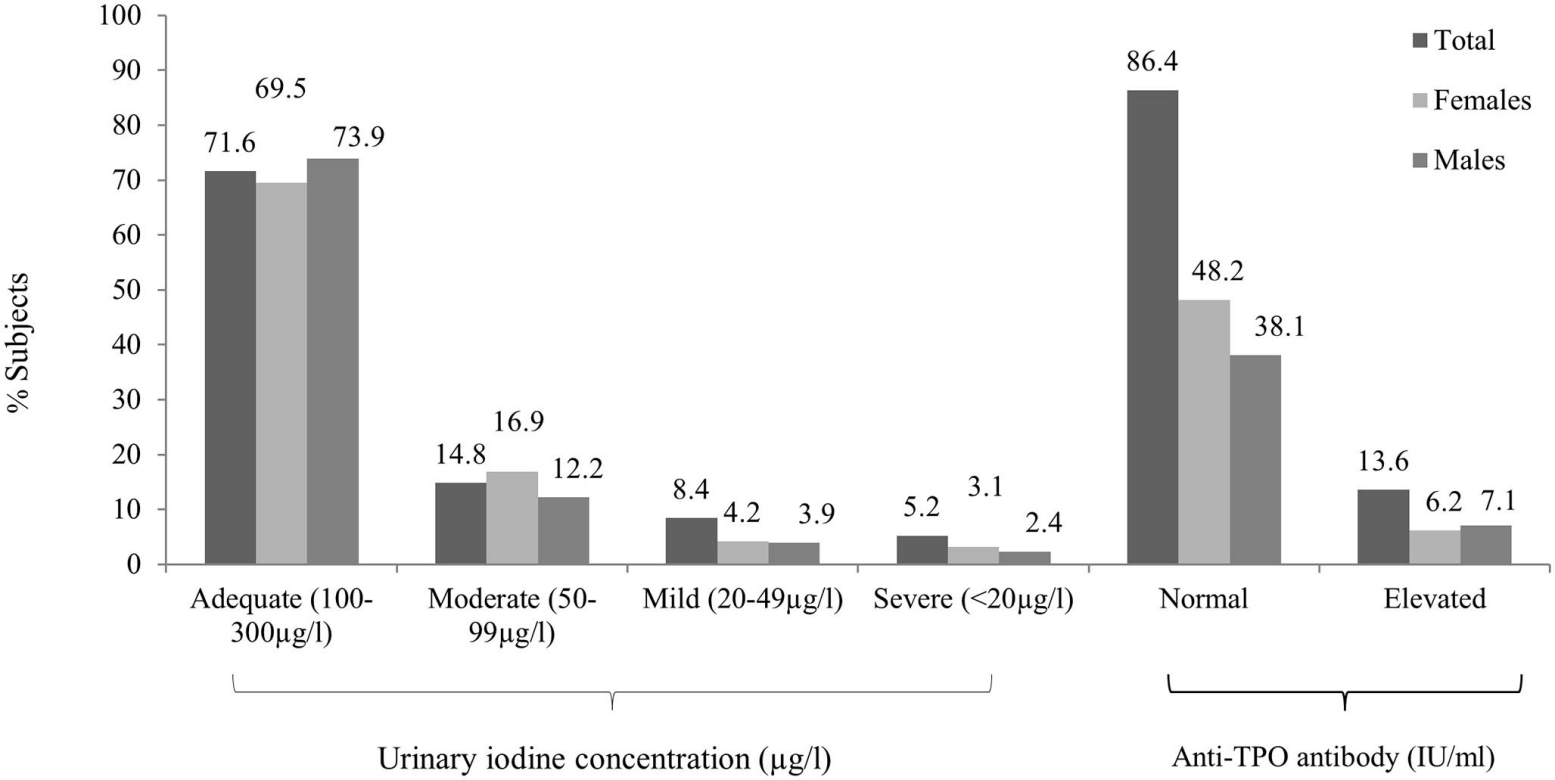
Overall and gender-wise distribution of urinary iodine concentration and antithyroid peroxidase (anti-TPO) antibody positivity between male and female subjects.

The median [interquartile range (IQR)] UIC was 154.50 (135) μg/L [156.13 (134) μg/L in men and 147.26 (136) μg/L in women]. ID, as defined by a UIC < 100 μg/L, was observed in 28.4% subjects (Figure 2). Around 14.8% subjects had mild deficiency, which was significantly more common in female subjects (*P* < 0.01). The remainder of the participants had UIC between 100 and 299 μg/L (Table 3). None of the participants had UIC ≥300 μg/L. ID was more common in elderly group as compared to others (Supplementary Table 1). UIC showed negative correlation with anti-TPO Ab (*r* = −0.08, *P* < 0.05) but no correlation with TSH. Further, multiple regression analysis revealed that age, gender, or UIC did not significantly affect TSH or thyroid dysfunction (Supplementary Table 2).

**Table 3:** Description of thyroid antibody and urinary iodine concentration among euthyroid vs. subjects with various thyroid disorders.

		**Hypothyroidism**		**Euthyroid**	**Hyperthyroidism**	
		**Overt** **n (%)**	**Subclinical** **n (%)**		**Subclinical** **n (%)**	**Overt** **n (%)**
Urinary Iodine excretion	763	52 (6.8)	184 (24.1)	510 (66.8)	10 (1.3)	7 (0.9)
Severe/moderate iodine deficiency (<50 μg/l)	104 (13.6)	8 (15.4)	25 (13.6)	68 (13.3)	2 (20)	1 (14.2)
Mild iodine deficiency (50–99 μg/L)	113 (14.8)	8 (15.4)	26 (14.1)	75 (14.7)	2 (20)	2 (28.6)
Optimal UIC iodine (100–300 μg/L)	546 (71.6)	36 (69.2)	133 (72.2)	367 (72)	6 (60)	4 (57)
Serum anti-TPO levels (IU/ml), n (%)	560	47 (8.4)	170 (30.4)	334 (59.6)	6 (1.1)	3 (0.5)
Normal	484 (86.4)	36 (76.6)	145 (85.3)	295 (88.3)	5 (83.3)	3 (100)
Elevated	76 (13.6)	11 (23.4)	25 (14.7)	39 (11.7)	1 (16.6)	0 (0.0)

## Discussion

Thyroid dysfunction, the most common endocrine disorder affecting the general population, contributes significantly to morbidity of general population. J&K is nested along the Himalayan region and is one of the known endemic areas of iodine deficiency diseases (11, 12). Tribal population residing in hilly areas is traditionally considered a high-risk group for iodine deficiency (1, 4). However, after universal salt iodization, we intended to know the current magnitude of iodine deficiency and its impact on the prevalence of thyroid dysfunction and autoimmunity in this underprivileged community.

A study conducted two decades ago among school-going children from non-tribal population in Kashmir region revealed the mean UIC of 49.6 ± 3.6 μg/g of creatinine (5). Another follow-up study 15 years later in the same region showed a remarkable improvement in the iodine status of Kashmiri schoolchildren with mean UIC of 123.6 ± 5.3 μg/L (6). Our study is the first to capture data regarding the iodine status of the tribal population of the J&K. In our study, the median (IQR) for UIC was 154.50 (135) μg/L, with no significant difference between male and female subjects (*P* = 0.09). It was comparable to our previous study done about a decade back in a tertiary care hospital among pregnant women, where we found a mean UIC of 143.78 ± 17.65 μg/L in euthyroid women in the first trimester; although still suboptimal, it revealed that iodine status relatively improved when compared to earlier reports from the region (13). This could be attributed to increased awareness and use of iodized salt over the last decade.

Despite adequate iodine intake (overall), this survey revealed evidence of ID in some areas. UIC being a measure of population iodine status rather than that of individuals, the extrapolated data from this small study may furnish preliminary insights into the extent of residual deficiency in the post-iodization era in the tribal subjects of J&K. The overall prevalence of ID in our study is 28.4%, while the 1995 survey in Kashmir valley revealed that 50% of school-going children had low UIC suggestive of ID (5). Although the prevalence seems to have come down by almost half in 25 years of universal iodine supplementation, it is still far higher than that reported (12.7%) by us among the residents of Delhi (8). This persistent high prevalence of ID in this underserved population may be due to consumption of non-iodized salt, which is still prevalent among some tribal areas of the J&K. However, the scenario looks better than the very high prevalence of ID found in other tribal communities in India like the Niyamgiri mountain area, Orissa (51.7%) and Kerala (87.5%), due to consumption of non-iodized salt (9, 10).

Anti-TPO antibodies were found to be positive in 13.5% patients, which is similar to the observations reported in non-tribal subjects (7, 8) across India. We observed a rise in anti-TPO positivity with age as evidenced by the fact that 9.2% of the subjects below 18 years vs. 20.3% of elderly population were anti-TPO Ab positive. TPO Ab positivity was also found to be higher in women as compared to men (7.1 vs. 5.2%). In our previous study, thyroid TPO Ab was mildly positive (>34 but <102 IU/ml) in 13.3% adults and strongly positive (i.e., >102 IU/ml) in 6.1% men and 10.8% women (8). Another Indian study among adults with long-term iodine sufficiency (median UIC = 220 μg/L) in the South Indian state of Kerala showed a prevalence of 16.7% anti-TPO positivity (14). In the literature, there are some studies that found higher prevalence of autoantibodies in iodine-deficient areas from Brazil and Denmark (15, 16), while others found low prevalence of antibody positivity in iodine-deficient areas from Nigeria (17). This may be due to difference in ethnicity and genetic predisposition to formation of autoantibodies among different populations. Besides, prevalence of anti-TPO Ab positivity may be affected by various other factors including analytic variations like assay variability, lack of international standards, and use of different cutoffs to define positivity in different studies.

We found that thyroid dysfunction was present in 33.2% of patients (36.3% male and 30.5% female). Among the thyroid disorders, SCH was the most common thyroid abnormality detected (24.1% of subjects), being more common in female (26.2%) as compared to male subjects (22.4%). A study done a decade back in a tertiary care hospital in Kashmir involving 2,550 subjects revealed a prevalence rate of SCH as 21.6% (18). Another hospital-based study on non-tribal population of Kashmir valley (19) revealed the prevalence of SCH to be 33%, with more number of female affected than male subjects (35 vs. 27.5%). These figures are higher than those reported from other parts of India (1, 7, 8). In our study on non-tribal population from Delhi (8), SCH was found to be lower (19.3%). Another study from South India (14) revealed a prevalence of SCH to be 9.4% in iodine-sufficient belt in Cochin (11.4% women vs. 6.2% men). The studies from areas with borderline to moderate iodine deficiency, including a report from the Indian state of Gujarat, show a lower prevalence of SCH ranging from 1.8 to 7% (20–22), while as the prevalence from areas transitioning to an iodine-sufficient state have been shown to vary between 4.9 and 10.4% (23). Similarly, higher prevalence (36.6%) of SCH was seen in tribal women from Godavari district in Andhra Pradesh (24). In iodine-replete areas, most people with thyroid disorders have autoimmune disease. The much higher prevalence of SCH than thyroid autoimmunity reveals ID to be a common cause of such thyroid dysfunction in our study. The prevalence of overt hypothyroidism is similar to one reported from various studies in different parts of India (3.9–4.2%) and probably reflecting the lesser severity of ID to produce frank hypothyroidism (8, 14).

The prevalence of thyrotoxicosis was similar to other large prevalence studies from India where both overt and subclinical hyperthyroidism were found in 1.1% (8). In another epidemiological study from Cochin, subclinical and overt hyperthyroidism were present in 1.6 and 1.3% of subjects participating in a community survey (17). These values, however, are not significantly different from other population-based studies reported in the literature (25, 26). Subclinical thyrotoxicosis was found to be more common in tribals from Kerala (3.2%) than (1.3%) in our study (16). Thyroid autoimmunity was almost similar in both studies (13.6 vs. 10.6%). However, ID was more prevalent in Kerala as compared to our study (87.5 vs. 28.4%), and improved iodine status in the background of goiter and higher prevalence of ID could have resulted in thyrotoxicosis. Autoimmunity can develop in nodular goiters leading occasionally to thyrotoxicosis, and iodization programs can also induce thyrotoxicosis, especially in those aged more than 40 years with nodular goiters (2). There are a few limitations to the present study. First, we should have assessed the type of salt consumed and iodine content of salt that would provide better judgment about the success of iodization programs in real-life scenario. Second, information about clinical indicators of iodine status like prevalence of goiter was available in small number of subjects. Third, other factors that independently affect thyroid function and autoimmunity like smoking, micronutrient deficiency, goitrogen intake, etc. should have been assessed.

Notwithstanding these limitations, this is the first data on UIC and thyroid status among tribal population from J&K suggesting that ID is still prevalent in this subpopulation. This may mandate the need for renewed efforts to reinforce focus on remote rural area inhabiting tribals.

This preliminary data on iodine and thyroid status show a high prevalence of ID and SCH among tribal population of J&K. Education and effective communication strategies to community and different stakeholders are essential to increase the awareness on iodine nutrition, especially in these marginalized populations.

## Data Availability Statement

The data will be made available upon reasonable request to the corresponding author.

## Ethics Statement

The studies involving human participants were reviewed and approved by Institute Ethics Committee, Sher-I-Kashmir Institute of Medical Sciences, Srinagar, India. Written informed consent to participate in this study was provided by the participants' legal guardian/next of kin.

## Author Contributions

MG and BC designed the research. TS, MB, and SS analyzed the data and drafted the manuscript. SA, MN, and AY conducted the experiments. All authors contributed to the article and approved the submitted version.

## Conflict of Interest

The authors declare that the research was conducted in the absence of any commercial or financial relationships that could be construed as a potential conflict of interest.
