# Supplementary material for: Thyroid Function, Urinary Iodine, and Thyroid Antibody Status Among the Tribal Population of Kashmir Valley: Data From Endemic Zone of a Sub-Himalayan Region
Source: Front Public Health. 2020 Oct 28;8:555840. doi: 10.3389/fpubh.2020.555840 (PMC7655871; doi:10.3389/fpubh.2020.555840)
Supplement: Supplementary file 1 [file Table_1.DOCX]

Supplementary Table 1: Showing status of thyroid auto antibody and urinary iodine excretion among age categories

|  | < 18 years | 18-60 years | > 60 years | Overall |
| --- | --- | --- | --- | --- |
|  | n (%) | n (%) | n (%) | n (%) |
| Urinary Iodine excretion (µg/l) n (%) | 89 (11.5) | 583 (76.8) | 91 (11.7) | 763 |
| Severe deficiency | 3(3.4) | 30 (5.17) | 7(7.95) | 40(5.3) |
| Moderate Deficiency | 4 (4.5) | 51(8.79) | 9(9.9) | 64 (8.47) |
| Mild Deficiency | 7 (8) | 92 (15.8) | 14(15.9) | 113(15.0) |
| Adequate | 75 (84.2) | 410 (70.3) | 61(67) | 546(71.6) |
| Anti-TPO (IU/ml) n (%) | 65 (11.7) | 432(77.7) | 59(10.6) | 556 |
| -(negative) | 59 (90.7) | 374(86.6) | 47(79.7) | 480 (86.3) |
| + (positive ) | 6 (9.2) | 58(13.4) | 12(20.4) | 76(13.7) |
