# Supplementary material for: Thyroid Function, Urinary Iodine, and Thyroid Antibody Status Among the Tribal Population of Kashmir Valley: Data From Endemic Zone of a Sub-Himalayan Region
Source: Front Public Health. 2020 Oct 28;8:555840. doi: 10.3389/fpubh.2020.555840 (PMC7655871; doi:10.3389/fpubh.2020.555840)
Supplement: Supplementary file 2 [file Table_2.DOCX]

| **Variables** | **Sub clinical Hypothyroidism** | **Overt Hypothyroidism** | **Crude odd ratio (95% CI)** | **P - value** |
| --- | --- | --- | --- | --- |
| Gender |  |  |  |  |
| Male | 33 | 28 | 1(Ref) |  |
| Female | 35 | 29 | 0.977 (0.483 – 1.975) | 0.947 |
| Age |  |  |  |  |
| 0 – 20 | 7 | 7 | 1(Ref) |  |
| 21 – 40 | 28 | 23 | 1.21 (0.3726 – 3.977) | 0.74 |
| 41 – 60 | 20 | 22 | 0.9091 (0.2711 – 3.0489) | 0.87 |
| >60 | 11 | 4 | 2.75 (0.5828 – 12.976) | 0.20 |
| Urinary Iodine concentration |  |  |  |  |
| Mild Deficiency | 2 | 9 | 1 (Ref) |  |
| Moderate | 8 | 48 | 0.75 (0.1363 – 4.1271) | 0.74 |
| Adequate | 58 | - | - |  |

Supplementary Table 2: A multivariate analysis of TSH with age, gender and UIC in the study population
